# Drone-DETR: Efficient Small Object Detection for Remote Sensing Image Using Enhanced RT-DETR Model

**DOI:** 10.3390/s24175496

**Published:** 2024-08-24

**Authors:** Yaning Kong, Xiangfeng Shang, Shijie Jia

**Affiliations:** College of Computer and Communication Engineering, Dalian Jiaotong University, Dalian 116028, China; kyn@djtu.edu.cn (Y.K.); shxf@djtu.edu.cn (X.S.)

**Keywords:** UAV object detection, RT-DETR, small object detection, feature fusion, visual sensors

## Abstract

Performing low-latency, high-precision object detection on unmanned aerial vehicles (UAVs) equipped with vision sensors holds significant importance. However, the current limitations of embedded UAV devices present challenges in balancing accuracy and speed, particularly in the analysis of high-precision remote sensing images. This challenge is particularly pronounced in scenarios involving numerous small objects, intricate backgrounds, and occluded overlaps. To address these issues, we introduce the Drone-DETR model, which is based on RT-DETR. To overcome the difficulties associated with detecting small objects and reducing redundant computations arising from complex backgrounds in ultra-wide-angle images, we propose the Effective Small Object Detection Network (ESDNet). This network preserves detailed information about small objects, reduces redundant computations, and adopts a lightweight architecture. Furthermore, we introduce the Enhanced Dual-Path Feature Fusion Attention Module (EDF-FAM) within the neck network. This module is specifically designed to enhance the network’s ability to handle multi-scale objects. We employ a dynamic competitive learning strategy to enhance the model’s capability to efficiently fuse multi-scale features. Additionally, we incorporate the P2 shallow feature layer from the ESDNet into the neck network to enhance the model’s ability to fuse small-object features, thereby enhancing the accuracy of small object detection. Experimental results indicate that the Drone-DETR model achieves an mAP^50^ of 53.9% with only 28.7 million parameters on the VisDrone2019 dataset, representing an 8.1% enhancement over RT-DETR-R18.

## 1. Introduction

The versatility and maneuverability of unmanned aerial vehicles (UAVs) enable them to efficiently conduct wide-area object detection in various and demanding settings. With advanced high-definition vision sensors, UAVs have been widely utilized in numerous applications such as disaster rescue operations [1], urban traffic [2], aerial surveying [3], military reconnaissance [4], and other critical fields.

**Figure 1:**
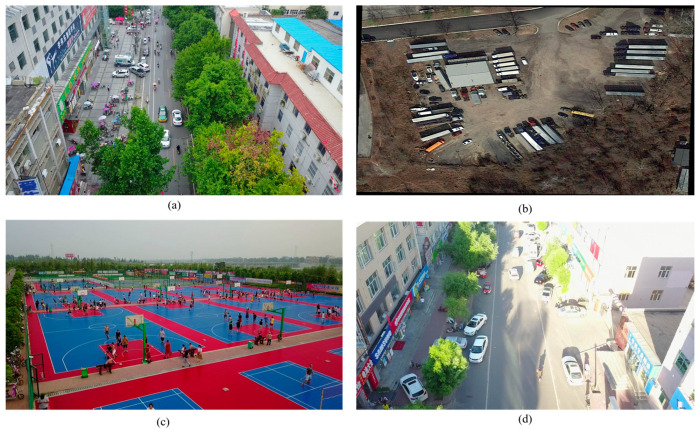
Sample images taken from a drone: (**a**) daytime aerial view of boulevard pedestrians and vehicles; (**b**) densely arranged trucks and cars; (**c**) crowd at a ballpark; (**d**) street scene with strong sun.

As depicted in Figure 1, UAV aerial images display notable distinctions when compared to ground-level images, characterized by several key features: (1) the image scene covers a relatively large area, including extensive background elements; (2) the primary shooting angle is from an overhead perspective, often resulting in occlusions; (3) there is a significant variation in object scale within the images—for example, a car in the foreground may appear up to five times larger than a car in the background; (4) individual object sizes are relatively small in relation to the overall image dimensions; (5) objects are frequently densely packed, with numerous similar items overlapping in certain scenes; and (6) various lighting effects, such as overexposure and insufficient illumination, are present in some images.

UAV object detection technology encounters two significant challenges. Firstly, in terms of model deployment, the constrained embedded resources of UAVs necessitate the utilization of lightweight real-time object detection models. This demand poses a crucial challenge in attaining an optimal equilibrium between model accuracy and processing speed [5]. Secondly, in the domain of aerial image recognition, UAV remote sensing images predominantly feature small objects with significant scale variations and diverse backgrounds. Additionally, visual sensors may introduce aberrations, causing objects to appear stretched or deflated to varying degrees. These factors pose significant challenges to object recognition and detection [6]. Therefore, the problem of object detection in UAV aerial images mainly lies in how to design a high-performance lightweight UAV small object detection model with low parameter count, high accuracy, and low inference delay.

Currently, object detection methods for UAV aerial images primarily use deep learning techniques. Deep learning-based object detection algorithms are mainly categorized into two-stage R-CNN improvement series [7,8,9,10,11] and single-stage YOLO series [12,13,14,15,16], as well as DETR improvement [17,18,19,20]. Among them, one-stage object detection techniques have significant advantages in real-time detection. Currently, Convolutional Neural Network (CNN)-based one-stage object detection techniques demonstrate good performance under typical viewing angles, especially the improved algorithm after YOLOv5 [14], which achieves higher detection accuracy and faster detection speed in natural image detection through a series of improvements in the network model structure, activation function, loss function, network training strategy, etc. However, it does not perform well in the object detection of UAV aerial images. On the one hand, current CNN-based algorithms frequently utilize non-maximum suppression (NMS) for post-processing. In real-time detection tasks, when there are a large number of objects in the image, this will lead to a significant increase in computational complexity, potentially affecting the stability of detection speed. On the other hand, drone aerial images mostly consist of large-scale images captured from a bird’s-eye view with small objects, overlapping multi-scale objects, and complex backgrounds, which makes it difficult to capture the object details and spatial relationships by completely using a convolutional kernel, thus reducing detection accuracy. The Transformer [21] has a natural advantage over traditional CNN in processing sequential data by capturing global dependencies through multi-head self-attention, which enables it to better capture complex relationships between objects [22]. The ViT model [23] is the first model to use a pure Transformer structure for image classification, and it outperforms CNN networks when the amount of training data is large enough. The DETR (DEtection TRansformers) [17] model combines the advantages of traditional CNNs and Transformer models. It adopts a hybrid architecture (CNNs + Transformer), which enables DETR to fully utilize the advantages of CNNs in extracting image features and Transformer in capturing global dependencies. It predicts the location and class of the object directly through an end-to-end learning approach, eliminating the need for a traditional anchor and the NMS post-processing steps. This enables DETR to achieve higher detection accuracy and more stable detection speed in object detection tasks. However, the high computational effort of the DETR-like model limits its practical use. In order to address the real-time issue of the DETR model, the literature [20] introduces a real-time variant called RT-DETR, which outperforms the current state-of-the-art YOLO series model in terms of both detection accuracy and speed. The RT-DETR model utilizes CNNs (ResNets [24]) as the backbone for feature extraction and employs the Transformer encoder for global correlation in the last layer of the feature extraction network. A CCFM structure, similar to the PAFPN [25] structure, is established in the neck network, and in which the fusion module is utilized to merge the top-down and bottom-up feature maps. The dense prediction results from the fusion module are provided to the decoder for prediction after query selection [19] to select the top Top-K items.

In the RT-DETR model, the backbone network employs ResNet for feature extraction. In the realm of UAV aerial images, the downsampling module in the ResNet network loses a significant amount of information about small objects. This issue hinders the backbone network’s capacity to accurately capture the subtle characteristics of these small objects. According to the research in the literature [26], small objects mainly exist in shallow feature maps and are easily lost in the process of multi-level downsampling. Meanwhile, the literature [27] indicates that the feature maps are highly correlated with each other, and standard convolution has some redundant computation issues. To address the aforementioned issues, this paper designs the ESDNet backbone network, reduces the feature loss during the downsampling process through the designed hybrid pooling downsampling module MPD, and decreases redundant computations by incorporating the FasterNet Block [27]. Moreover, this paper proposes the shallow feature enhancement module (SFEM) to enhance the extraction capacity of shallow features. The output of the SFEM layer is directly integrated into the neck network for feature fusion, thereby enhancing the network’s capability to extract and represent small object features. At the same time, the network structure gradually reduces the number of deep feature maps that contribute less to small object detection, which improves the network’s lightweighting effect while maintaining accuracy.

In the neck network, the fusion module of RT-DETR is responsible for fusing multi-scale features. In UAV aerial images, the distribution and scale structure of the objects varies with different camera angles of the UAV. Current fusion modules only use simple residual concatenation and RepBlock [28] to fuse multi-scale features, where the standard convolution is limited when dealing with geometrically deformed objects. This may result in models that fail to adequately mine and express complex feature information. In order to overcome the limitations of the current fusion module, this paper designs the EDF-FAM module. This module fuses Dual-Path features and utilizes deformable convolution [29] and 1D-channel convolution with three different convolution kernel sizes to construct a global attention mechanism for multi-scale feature maps. Compared with the fusion module, the deformable convolution in the EDF-FAM module can better adapt to the spatial variation of the objects, and have lower computational complexity.

In summary, the main improvements and innovations of this paper are as follows:

1. The lightweight backbone network ESDNet for UAV small object detection has been designed. Within it, a shortcut downsampling module MPD, which utilizes MaxPool and AvgPool together, is incorporated. Furthermore, a Fast-Residual Block is proposed, based on MPD and the FasterNet Block. Additionally, a shallow feature enhancement module (SFEM) consisting of C2f and the Fast-Residual Block is also introduced. This significantly enhances the shallow feature extraction capability and achieves model lightweight simultaneously.
2. An Enhanced Dual-Path Feature Fusion Attention Module (EDF-FAM) has been developed to collaboratively generate global attention from dual-channel features through the integration of deformable convolution and 1D-channel convolution. This multi-scale fusion strategy facilitates the interaction of multi-channel information, thereby significantly improving the feature representation capabilities of the model.
3. The output of the SFEM layer is introduced into the neck network for feature fusion on the basis of the original fusion network, which enhances the ability of the network to extract and express small object features.

## 2. Related Work

### 2.1. Drone Object Detection Technologies

The development of UAV object detection technology has made remarkable progress in recent years. With the rapid development of computer vision and deep learning technology, object detection algorithms have been widely used in UAV applications. Traditional object detection algorithms are primarily based on image processing and feature extraction, but they have limitations in complex scenes. The object detection algorithm based on deep learning, on the other hand, can detect the object more accurately and in real time. With the advancement of deep learning, object detection algorithms for UAVs are emerging. Zhang et al. [30] proposed a ViT-YOLO algorithm for small object detection, which enhances the spatial feature extraction capability of the backbone network by incorporating a multi-head self-attention layer (MHSA). However, the detector head of the model utilizes a maximum of 128-fold downsampling, leading to increased computational complexity and parameter count. In the detection of small objects, it has been demonstrated that the 64-fold and 128-fold downsampling feature maps contribute less to the average precision (AP). Therefore, while ViT-YOLO excels in certain aspects, it still faces challenges due to high computational complexity and the phenomenon of “diminishing marginal benefit”. Zhu et al. [31] proposed an improved YOLOv5 algorithm for UAV images. TPH-YOLOv5 utilized Transformer Prediction Heads (TPH) to replace the original detection head to explore the prediction potential with a self-attention mechanism. In addition, they integrated the Convolutional Block Attention Model (CBAM) in order to find regions of attention in object-dense scenes, thereby enhancing the detection performance. However, TPH-YOLOv5 utilized multiple transformer modules in the backbone and neck networks. This leads to poor real-time inference performance and high computational complexity of the model. Zhang et al. [6] introduced a drone detection algorithm, Drone-YOLO, which enhances YOLOv8 by efficiently integrating shallow features in the neck network through a sandwich structure, thereby enhancing the accuracy of small object detection. Li et al. [32] proposed a low-parametric object detection algorithm using an improved YOLOv8-s model. This model replaces the PAFPN structure in the neck network with Bi-FPN and incorporates Ghost blocks into the backbone network to achieve smaller parameters and higher detection accuracy. Deng et al. [26] proposed an Extended Feature Pyramid Network (EFPN) for small object detection, which provides feature mapping of the shallow backbone network through the designed Feature Texture Transfer (FTT) module and directly to the feature fusion network, achieving the highest accuracy on the MS COCO dataset.

### 2.2. Transformer-Based Object Detection Network

In the field of object detection, the application of Transformer models is also gradually attracting attention. The Vision Transformer (VIT) [23] demonstrated for the first time that the Transformer architecture can also be directly applied to image processing by decomposing an image into multiple patches. Its performance on image recognition tasks is comparable to that of state-of-the-art convolutional networks. It is worth noting that DETR [17] is the first approach to successfully utilize Transformers for object detection techniques. Specifically, DETR utilized standard CNN models (e.g., ResNet50/101) for feature extraction and image decomposition. The extracted features are then inputted into a Transformer encoder, which subsequently produces object location and category information through a decoder. Finally, DETR optimizes the parameters of the model through end-to-end training so that it can accurately detect objects in the image. The DETR model can achieve true end-to-end training and inference without any post-processing (NMS) operation through the global self-attention mechanism, but there are still two major Transformer architecture problems that lead to long training cycles, slow convergence, and poor detection of small objects. To address these issues, Deformable-DETR [18] introduced the deformer attention module, which converts the densely connected attention module into a trainable sparse attention module, thereby significantly enhancing the convergence speed. However, the computational cost of the decoder is still a bottleneck. Efficient DETR [33] enhances the query capability of the decoder by selecting the top K positions from the dense predictions of the encoder. However, while the aforementioned models enhance the convergence speed and detection accuracy, they overlook the escalating computational complexity that results from numerous stacked encoders and decoders. This makes it challenging to meet the real-time requirements. RT-DETR [20] found that the final layer in the feature map extracted by CNN contains most of the global information. Using the encoder module only for the last layer can greatly improve the inference speed without decreasing the accuracy. The encoder feature map is fused with shallow multi-scale features to form a feature pyramid. Subsequently, the enhanced query selection is provided to the decoder for prediction. By utilizing only one layer of the encoder, the computation load is significantly reduced, which gives RT-DETR obvious advantages in accuracy and speed. Therefore, RT-DETR is chosen as the baseline model in this paper.

## 3. Methodology

**Figure 2:**
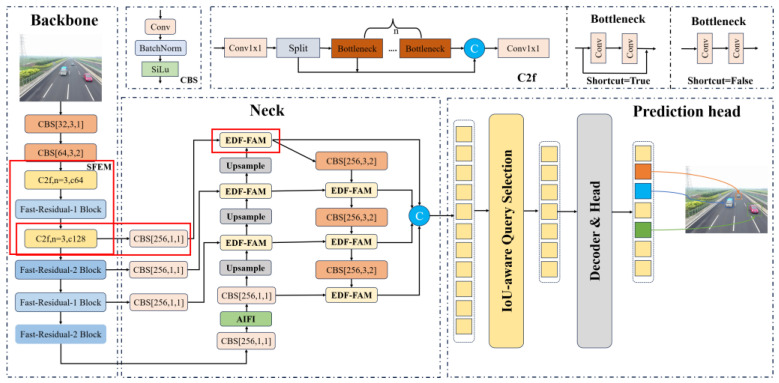
Structure of Drone-DETR.

The architecture of Drone-DETR is illustrated in Figure 2. The first component consists of the lightweight small object detection backbone network, ESDNet, which introduces the Fast-Residual Block as a lightweight feature extraction module. Additionally, the shallow feature enhancement module (SFEM) is constructed using the C2f module [16] alongside the Fast-Residual Block. The C2f module, inspired by the ELAN concept from YOLOv7 [15], ensures a lightweight design while facilitating a richer gradient flow, thus enhancing feature extraction efficiency. A detailed description of ESDNet can be found in Section 3.1. In the second component, an additional downsampled layer with a 4-fold reduction is integrated on top of the original feature map extraction from the backbone network, aimed at extracting higher-quality spatial and channel features. This enhancement significantly improves the recognition of small and tiny objects, as discussed in the first half of Section 3.2. The third component introduces the Enhanced Dual-Path Feature Fusion Attention Module (EDF-FAM), which replaces the fusion module of the neck network. This module efficiently aggregates features from various backbone layers. A detailed discussion is provided in the second half of Section 3.2. Finally, the dense prediction results produced by the EDF-FAM module are forwarded to the decoder for prediction. This process involves query selection [19] to identify the top K items for prediction.

### 3.1. Backbone

**Figure 3:**
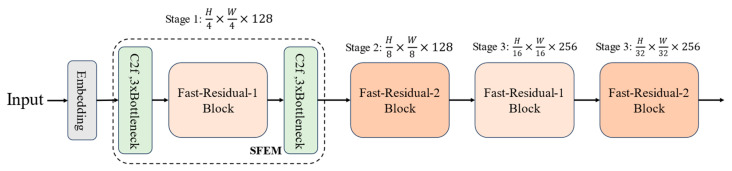
Structure of ESDNet.

**Figure 4:**
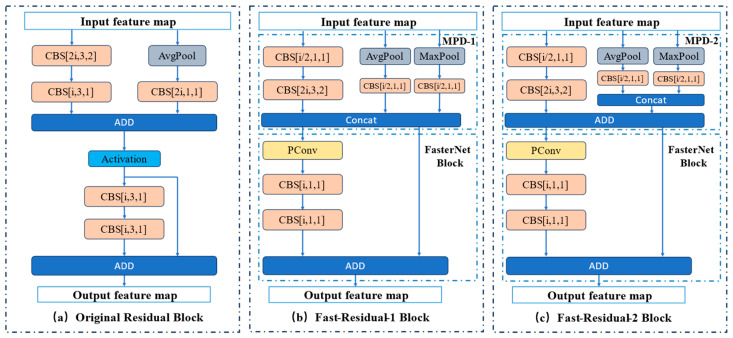
Comparison of the Fast-Residual Block and Residual Block.

**Figure 5:**
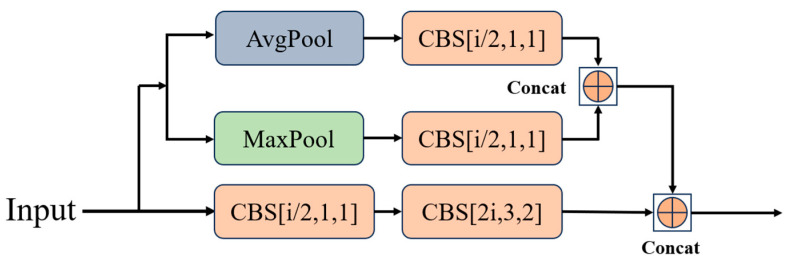
MPD-1 module structure.

**Figure 6:**
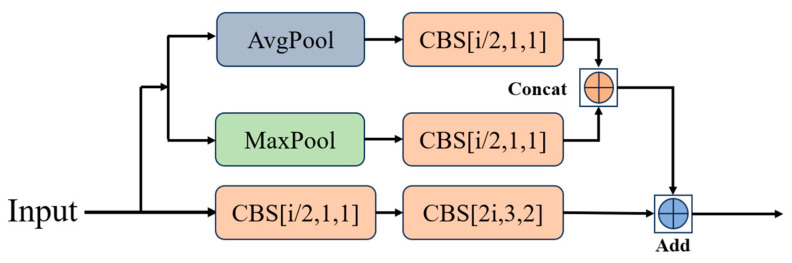
MPD-2 module structure.

**Figure 7:**
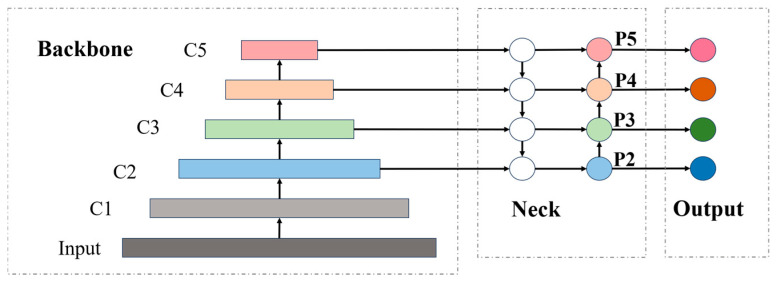
P2-enhanced layer and PAFPN structure in Drone-DETR.

The overall architecture of ESDNet is illustrated in Figure 3. The general structure of the backbone is similar to that of ResNet [24], which ultimately performs 32-fold downsampling. To minimize the impact of downsampling on feature extraction, the initial embedding layers consist of a 3 × 3 convolution with a stride of 1, followed by a 3 × 3 convolution with a stride of 2. The feature maps are then downsampled to 320 × 320. The P2 detection layer uses the C2f Module [16] and Fast-Residual Block to construct a shallow feature enhancement module (SFEM) to improve the model’s multi-scale sensing capability. The P3 detection layer utilizes the Fast-Residual-2 Block for feature extraction. It downsamples feature maps to (80 × 80) while decreasing the number of convolutional kernels for subsequent deep feature maps that have less impact on small object detection. The P4 and P5 detection layers employ the Fast-Residual-1 Block and Fast-Residual-2 Block for feature extraction, restricting the number of convolutional kernels to 256. For details of each block, please refer to the detailed structure of each block in Figure 2, Figure 4, Figure 5, Figure 6 and Figure 7.

#### Fast-Residual Block

Figure 4 illustrates a comparison between the Fast-Residual Block and the Residual Block, where “i” represents the number of input channels per layer. Figure 4a shows the original residual module, which consists of a set of downsampled residual blocks and a set of original residual blocks utilizing regular convolutional layers. Figure 4b shows Fast-Residual-1 Block replacing the downsampled residual block with MPD-1 Block and the raw residual block with FasterNet Block [27]. MPD-1 Block consists of a downsampled convolutional layer and a pooled residual join using average pooling and maximum pooling together to construct the shortcut. FasterNet Block consists of a PConv layer [27] and two 1 × 1 Conv layers to form the residual structure, and PConv performs convolution operations on only part of the feature map.

The difference between Fast-Residual-2 Block and Fast-Residual-1 Block lies in the downsampling module. The pooled residual layer of the MPD-2 module is connected using ‘Concat’ as in the MPD-1 module, the difference being that the downsampling convolutional layer and pooled residual layer are connected using Add, which makes it possible for the MPD-2 module to keep the inputs and outputs consistent.

The two MPD modules are shown in Figure 5 and Figure 6. The MPD module is used in the Fast-Residual Block for downsampling and channel expansion. Use MaxPool and AvgPool together to build the shortcut. Additionally, the traditional convolutional feature extraction process is optimized by reducing the number of channels using 1 × 1 Conv before conducting the downsampling operation.

### 3.2. Neck

As depicted in Figure 7, in the backbone network, the receptive field’s range can be extended by continuously stacking the convolutional layers. The expansion of the overlap area between the receptive fields results in further compression of image information, facilitating the acquisition of comprehensive image details. However, as spatial information is continuously compressed during the downsampling process, it becomes easy to lose details of small objects due to oversampling. This can make it challenging to extract features of small objects. To address this issue, this paper introduces the shallow feature layer of SFEM in the ESDNet network to offer high-quality feature mapping into the upper and lower branches of PAFPN [25] in the neck network. The features primarily originate from the shallow features, which preserve a significant amount of fine-grained information on small objects, thereby enhancing the performance of small-object detection.

**Figure 8:**
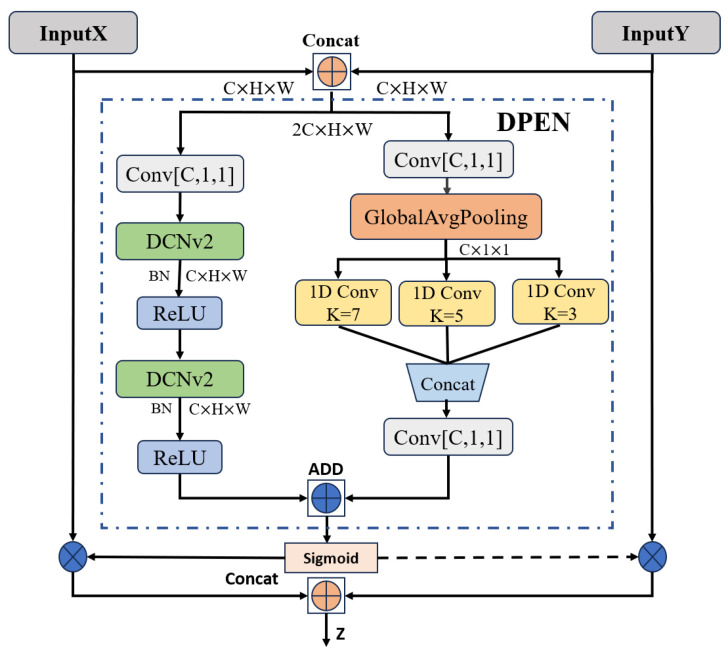
Structure of EDF-FAM.

As shown in Figure 8, in this paper, we designed an Enhanced Dual-Path Feature Fusion Attention Module (EDF-FAM) for fusing multi-scale feature maps in neck networks. EDF-FAM consists of a Dual-Path Feature Extraction Network (DPEN) and dual primitive mapping. The DPEN structure includes a spatial and channel attention mechanism module that incorporates left and right channels for feature extraction. The left channel is utilized for spatial feature extraction through deformable convolution (DCNv2 [29]). The right channel is utilized for feature extraction through multiple 1D channel convolutions for adjacent sections of the feature map. The fused weighted points are finally multiplied back into the corresponding feature maps.

The spatial attention formula L(K) for global features is shown in Equation (1), and the spatial features are extracted by deformable convolution:

(1) $$L(X⊕Y)=\delta (B(DCNv2(\delta (B(DCNv2(Conv_{1\times 1}(X⊕Y)))))))$$

L(X⊕Y) denotes the summation of X and Y (Y denotes the feature map obtained after downsampling) features by channel. $Conv_{1\times 1}$ is a 1 × 1 convolution, which reduces the number of channels of the input feature to half of the original. ⊕ denotes the channel dimension concatenation superposition, B denotes the BatchNorm layer, and δ denotes the ReLU activation function. Feature extraction is performed on the feature map by DCNv2, and the offset is constantly updated to enhance the model fitting ability; this step does not change the number of output channels.

The channel attention formula R(K) for global features is shown in the following Equations (2) and (3), which examine the correlation between features at different scales through multiple sets of 1D convolutions:

(2) $$G(X⊕Y)=GAP(\delta (B(Conv_{1\times 1}(X⊕Y))))$$

(3) $$\begin{array}{cc} R(X⊕Y)= & Conv_{1\times 1}(1DConv_{k=7}(G(X⊕Y))⊕\\ & 1DConv_{k=5}(G(X⊕Y))⊕1DConv_{k=3}(G(X⊕Y))) \end{array}$$

The features are extracted after using $Conv_{1\times 1}$ to reduce the number of channels to half of the original count, and global average pooling (GAP) denotes the global average pooling layer, which inputs the globally averaged pooled feature maps as input into 1D convolution with kernels of sizes 7, 5, and 3, respectively. These are then superimposed based on the channel dimensions, and the channels are restored to the original count using $Conv_{1\times 1}$ to half of the original input channels.

The overall EDF-FAM computation is shown in Equations (4) and (5), where the spatial and channel attention feature maps are summed using a broadcast mechanism. Subsequently, after applying a sigmoid activation function, the output values are constrained to the range of 0 to 1. The symbol ⊗ denotes element-wise multiplication of the corresponding elements in the two feature maps. Attention weights are dynamically allocated to the feature maps of X and Y using (1−M(X⊕Y)), and the resulting outputs are combined along the channel dimension after this operation.

(4) M(X⊕Y)=Sigmoid(L(X⊕Y)+R(X⊕Y))

(5) Z=M(X⊕Y)⊗X⊕(1−M(X⊕Y))⊗Y

## 4. Experiments and Results

### 4.1. Dataset

**Figure 9:**
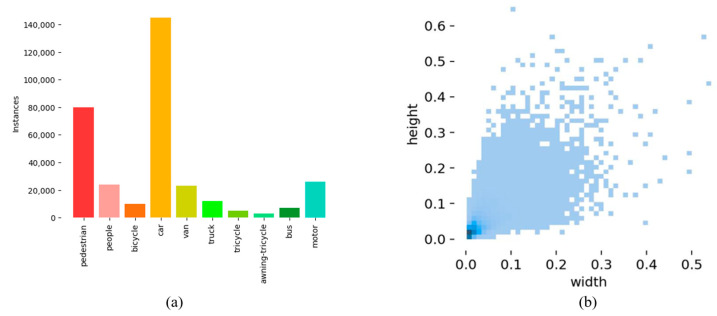
VisDrone2019 dataset: (**a**) Instances. Information of 10 classes in Visdrone-2019; (**b**) Proportion. The proportion of object size in VisDrone-2019, whose height and width are assumed to be 1.

The dataset used for the experiments in this paper is the VisDrone2019 public dataset [34]. Among them, 6471 are in the training set, 548 are in the validation set, and 1610 are in the test set. The images captured by UAVs have distinctive features, including significant size variations, complex environments with various disturbances, and diverse object shapes that are flexible and variable. The detailed description of this dataset is shown in Figure 9. As depicted in (a), the dataset comprises a total of 10 categories: pedestrian, people, bicycle, car, van, truck, tricycle, awning-tricycle, bus, and motor. The number of entities in each category varies significantly, with pedestrians and cars occupying the majority of the total, reflecting the transportation situation accurately. As shown in (b), most of the objects are smaller than 0.01 times the image size, indicating a large number of small and tiny objects in this dataset.

### 4.2. Experimental Environment

During the training process, the input image size is set to 640 × 640, the batch size is set to 4, and the number of epoch is set to 200. In this paper, the hyperparameters are based on RT-DETR, and the first 300 coded features are selected to initialize the object query module of the decoder. Our detector is trained using the AdamW optimizer base_learning_rate=0.0001, weight_decay=0.0001, global_gradient_clip_norm=0.1, linear_warmup_steps=2000, and a minimum learning rate of 0.00001. The experimental environment is illustrated in Table 1.

### 4.3. Experiment Metrics

In order to accurately evaluate the improvement effect of the proposed Drone-DETR, we utilize mean average precision (mAP), number of model parameters (Params), giga floating-point operations per second (GFLOPs), and frames per second (FPS) as the evaluation metrics for model performance. The details are listed below.

Precision (P) denotes the proportion of correctly predicted samples in the positive sample set. It is calculated through Equation (6), where TP represents the correct prediction objects, and FP represents the incorrect prediction objects.

(6) $$P=\frac{\mathrm{TP}}{\mathrm{TP}+\mathrm{FP}}$$

Recall (R) indicates the proportion of samples that are actually positive and predicted correctly. It is calculated through Equation (7), where FN represents objects that exist but have not been correctly detected.

(7) $$R=\frac{\mathrm{TP}}{\mathrm{TP}+\mathrm{FN}}$$

The average precision (AP) is a measure of the precision score at various thresholds along the precision–recall (PR) curve, calculated using Equation (8). The mean average precision (mAP) is the average of all classes of AP, which is derived via Equation (9). In order to better represent the model’s detection effect on objects of different sizes, our method evaluates the model using the mAP^50^, mAP^50–95^, mAP^S^, mAP^M^, and mAP^L^ metrics. Specifically, mAP^50^ represents the mAP when the IOU is 0.5, and mAP^50–95^ is the average mAP when the IOU ranges from 0.5 to 0.95. mAP^S^, mAP^M^, and mAP^L^ are used to evaluate objects of different sizes. On the basis of mAP^50^, for objects smaller than 32 × 32 pixels, mAP^S^ is used as an evaluation metric; for objects with pixels between 32 × 32 and 96 × 96, mAP^M^ is used as an evaluation metric; and for objects with pixels larger than 96 × 96, mAP^L^ is used as an evaluation metric.

(8) $$\mathrm{AP}=\int_{0}^{1}p(r)dr$$

(9) $$\mathrm{mAP}=\frac{1}{k}\sum_{i=1}^{k}{\mathrm{AP}}_{i}$$

### 4.4. Comparative Experimental Results and Analysis

#### 4.4.1. Comparative Experimental Results and Analysis of Drone-DETR and Other SOTA Algorithms

As shown in Table 2, this paper compares Drone-DETR with other SOTA algorithms, including two-stage algorithms such as CornerNet [35], ARFP [36], DMNet [37], DSHNet [38], and CRENet [39], and one-stage algorithms such as TPH-YOLOv5 [31], DMA-YOLO [40], Drone-YOLO [6], and YOLO-DCTI [41]. Some of the object detection algorithms in the table do not give fps because the algorithms are not real-time. In comparison with the typical one-stage detection algorithm YOLO-DCTI, the approach presented in this paper enhances the detection performance by 4.1% and 6.5% for mAP^50^ and mAP^50–95^, respectively. In addition, the algorithm presented in this paper achieves the highest mAP^50–95^ (33.9%) in nearly all comparisons. Although Drone-DETR slightly lags behind CRENet in mAP^50^, it still delivers a suboptimal result while offering a speed advantage. Compared with advanced one-stage generalized algorithms, some of the improvements made in this paper to enhance small object detection accuracy increase the computational complexity, which inevitably slows down the inference speed. However, the increase in computational complexity is worthwhile for the improvement in AP, while still maintaining real-time detection at the required FPS. The experimental results demonstrate that Drone-DETR can rapidly and efficiently recognize and accurately locate small objects in complex environments within UAV aerial images.

#### 4.4.2. Comparison of Drone-DETR and RT-DETR for Small Object Visualization

**Figure 10:**
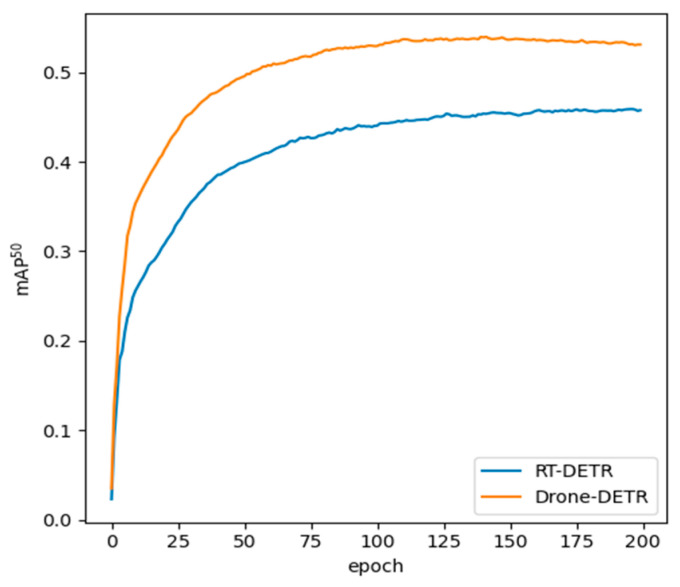
Visualization of mAP^50^ variations during training by Drone-DETR and RT-DETR.

In order to showcase the detection effect of Drone-DETR on small objects compared to the original model, the mAP^50^ of the validation dataset results during the training process is visualized, as depicted in Figure 10. Drone-DETR converges faster in the initial stage of training (first 25 epoch), and its mAP^50^ is higher than that of RT-DETR. As the training progresses (during the first 100 epoch), the performance advantage of the Drone-DETR model is further enhanced, and its mAP^50^ metrics continue to increase, while the performance of the RT-DETR model improves at a slower pace. This indicates that the Drone-DETR model demonstrates superior learning ability and detection accuracy during the training phase.

As depicted in Table 3, on VisDrone2019-test, Drone-DETR enhanced the detection performance of mAP^S^, mAP^M^, and mAP^L^ by 6.2%, 5.9%, and 5.8%, respectively, compared to RT-DETR. This suggests that Drone-DETR detection capability has improved across various scales, with a more noticeable enhancement in detecting small-scale objects.

In order to further validate the effectiveness of Drone-DETR in small object detection for UAV aerial photography, we conducted supplementary experiments on the DOTA dataset [44]. As shown in Table 4, the detection performance of Drone-DETR is significantly improved over RT-DETR on the DOTA validation set. Among them, mAP^S^, mAP^M^, and mAP^L^ were improved by 3.0%, 1.2%, and 0.6%, respectively. This result fully demonstrates the advantages of Drone-DETR in small object detection tasks and provides support for its effectiveness in practical applications.

**Figure 11:**
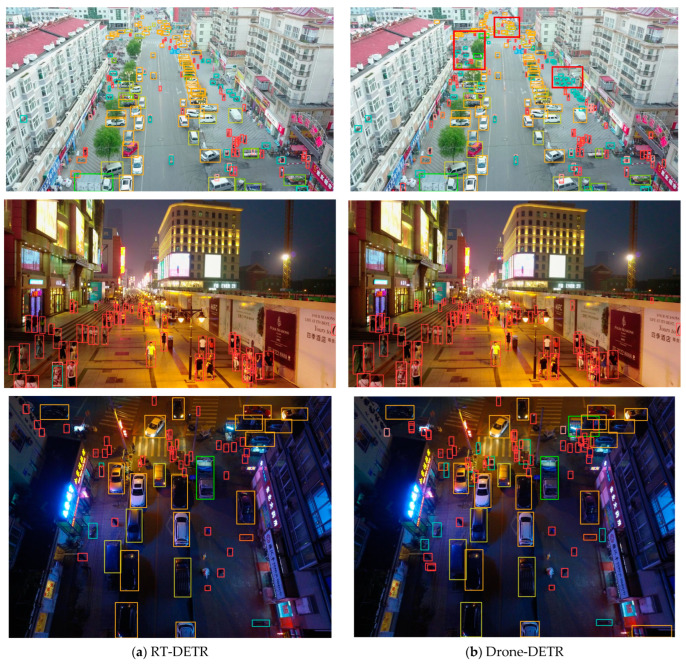
Visualization results of RT-DETR and Drone-DETR on VisDrone2019-test.

Figure 11 illustrates the comparison results of the model RT-DETR and Drone-DETR on VisDrone2019-test. Figure 11a shows the visualization results of RT-DETR before improvement, while Figure 11b displays the visualization results of Drone-DETR. Different colored borders are used to indicate various categories. In the high-density scenario, the improved model has significantly improved the detection performance of small objects, as depicted in the first row of Figure 11. It better recognizes small objects from a distance and more accurately identifies object categories up close. The second row demonstrates that our Drone-DETR model presented can effectively detect small objects at a distance in a scene with a strong light source. The third line demonstrates that the model proposed can still identify more valid objects in scenes with darker light sources.

#### 4.4.3. Results and Analysis of Fast-Residual Block vs. Residual Block

**Figure 12:**
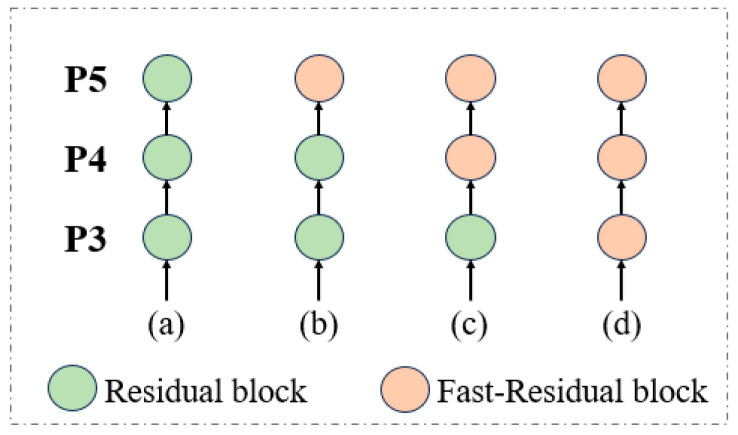
Experimental settings for comparing the Fast-Residual Block and Residual Block.

As shown in Figure 12, the experimental setup compares the Fast-Residual Block to the ResNet18 network. In this setup, the last three layers of the network are sequentially replaced with the Fast-Residual Block. The light green ball represents the Residual Block, while the light skin color represents the Fast-Residual Block.

In order to demonstrate the superiority of the Fast-Residual Block proposed in this paper, we utilized RT-DETR-R18 as the base network. Subsequently, the P5, P4, and P3 layers of ResNet18 were sequentially replaced with the Fast-Residual Block in a comparative test. The results of the comparative tests on VisDrone2019-Val are presented in Table 5. The comparison results are based on VisDrone2019-Val. From the results in Table 5, it can be seen that the accuracy of the model decreases after replacing the last layer of the Residual Block with the Fast-Residual Block. However, the accuracy of the model continues to increase, and the speed of inference accelerates as more shallow feature layers are replaced by the Fast-Residual Block. After replacing the last three layers entirely with Fast-Residual Blocks, the model’s accuracy increases by 0.1%, the number of parameters decreases by 18%, and the inference speed improves by 22%. The experimental results confirm the effectiveness of the Fast-Residual Block in enhancing the efficiency and performance of the model. It is noteworthy that the impact of the Fast-Residual Block becomes more significant when added earlier in the network. This effect may stem from the observation that adding the Fast-Residual Block earlier helps retain more downsampled features in the backbone network. This crucial enhancement plays a vital role in balancing accuracy and speed in the Drone-DETR network.

### 4.5. Results and Analysis of Ablation Experiments

The ablation experiment validates the algorithm’s performance with RT-DETR and Drone-DETR. The model is trained with consistent hyperparameters for 200 epoch. Subsequently, the model is evaluated on both the test and validation sets. There are four groups of models in total. Group A serves as the baseline model utilizing ResNet18 as the backbone network, with the input image size set at 640 × 640 pixels. Group B replaces the backbone network with ESDNet on Group A; Group C incorporates the EDF-FAM based on Group B; and Group D integrates the small object detection layer based on Group C.

Table 6 presents the experimental results of all the improvement strategies discussed in this paper on both the test and validation sets. Among them, Experiment B replaces the backbone network of the baseline model with ESDNet. As a result, the mAP^50^ increases by 1.1% on the test set, while the number of parameters decreases by 31.3% and the GFLOPs increase by 22.5%. Experiment C adds the multi-scale feature fusion attention module to Experiment B. Compared to the baseline model, the mAP^50^ increases by 3.5%, the parameters increase by 10.0%, the GFLOPs increase by 17.7%, and the FPS decreases by 11.9% over the test set. It is worth noting that in Experiment C compared to Experiment B, the GFLOPs for this improvement are lower than the computational complexity of the fusion module used in the original model. Experiment D adds the P2 small object feature layer based on Experiment C, which is derived from the shallow features of the backbone network. Compared with the baseline model, the enhanced model increases the mAP^50^ by 6.2% in the test set and by 8.1% in the validation set. Additionally, it decreases the FPS by 46. 

The experiment demonstrates that the ESDNet structure enhances object detection performance while reducing the number of parameters. Despite the increased complexity of the model, its enhanced feature extraction abilities result in higher accuracy metrics, setting the stage for potential future improvements. The EDF-FAM module efficiently focuses on key features and enhances object detection performance. By incorporating the shallow feature method, which provides crucial information for small object detection, into the feature fusion network, the model is effectively improved by integrating fine-grained details. This significantly enhances the performance of small object detection. However, the increase in model complexity inevitably affects operational efficiency, and the integration of shallow features still requires further optimization. It is important to note that with the substantial improvement in model accuracy and the increase in GFLOPs, the FPS performance of Drone-DETR remains within acceptable thresholds for real-time detection, with the total parameter count rising by a mere 8.6 million.

**Figure 13:**
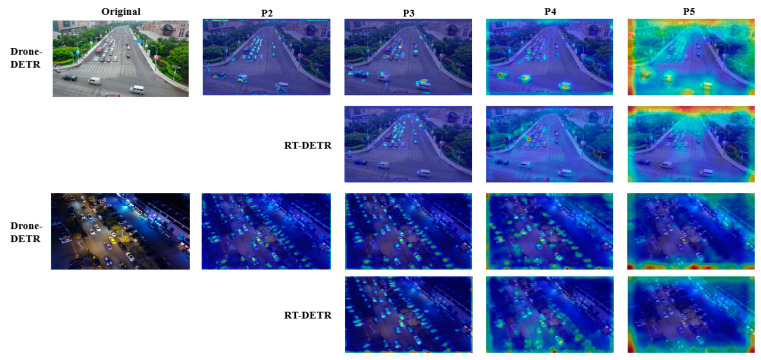
RT-DETR vs. Drone-DETR ROI region visualization results in different FPN fusion layers.

To demonstrate the effectiveness of incorporating the P2 layer and EDF-FAM into the neck network, our experiment conducts ROI visualization analysis of EDF-FAM feature layers from P2 to P5, generating heat maps for the original model and the model proposed in this paper. As shown in Figure 13, due to the different backbone network inputs accepted by EDF-FAM, the receptive field from layer P2 to P5 is expanding, and each layer does not specialize in recognizing the same size of objects. Layer P2 is more sensitive to small objects, while layers P3 and P4 pay more attention to medium objects. As can be seen in the figure, in the scenarios with a large number of small objects, layer P5 pays less attention to the objects, and it is more a kind of background attention. The EDF-FAM module can concentrate more weight on the object, effectively reducing interference from complex environments. It is obvious that the P2 layer can capture more detailed features compared to the P3 layer, indicating the crucial importance of the P2 layer in small object detection. Additionally, the global information of the P5 layer provides limited utility for detecting small objects, indicating that the lightweight enhancement of ESDNet is effective.

## 5. Conclusions

In this paper, a new enhanced UAV real-time object detector, Drone-DETR, is proposed based on RT-DETR. The SFEM module and Fast-Residual Block are constructed in the ESDNet backbone network to improve the model’s performance in small object detection and reduce computational costs. Additionally, an enhanced shallow feature layer is introduced in the neck network, replacing the fusion module with the EDF-FAM module proposed in this paper. The EDF-FAM module extracts multi-scale feature information and suppresses unnecessary background information through competitive learning. A large number of experiments on the VisDrone dataset and DOTA dataset show that the algorithm proposed in this paper has obvious advantages in balancing accuracy and speed. Especially for small object detection accuracy, it improves significantly compared with RT-DETR, and still meets the requirements of real-time detection. However, Drone-DETR also has some limitations. The detection performance is prone to degradation under certain conditions, such as lighting (exposure, dark light, etc.) and weather (fog, rain, etc.). In the future, we will continue to focus on research in the field of object detection in UAV aerial images, analyze the difficulties of detection, and propose more adaptable and lightweight models.

## Figures and Tables

**Table 1:** Experimental system environment.

Feature	Parameter
Operating System	Windows 10
Programming Language	Python 3.9
CPU	Intel(R) Xeon(R) W-2245 CPU @ 3.90 GHz
GPU	RTX 3090
GPU Memory	32 G
Algorithm Framework	PyTorch

**Table 2:** Performance comparison results of different models on VisDrone2019-Val.

Method	Reference	mAP^50–95^/%	mAP^50^/%	FPS
Two-stage methods				
CornerNet [35]	(Law et al., 2018)	17.4	34.1	
ARFP [36]	(Wang et al., 2022)	20.4	33.9	
DMNet [37]	(Li et al., 2020)	29.4	49.3	
DSHNet [38]	(Yu et al., 2021)	30.3	51.8	
CRENet [39]	(Wang et al., 2020)	33.7	54.3	
One-stage methods				
FRCNN [8] + FPN [42]	(Lin et al., 2017)	21.8	41.8	17
YOLOv5m [14]	(Jocher et al., 2022)	21.9	42.3	85
YOLOv7 [15]	(Wang et al., 2023)	23.0	41.1	55
TPH-YOLOv5 [31]	(Zhu et al., 2021)	23.1	41.5	25
YOLOv8m [16]	(Jocher et al., 2023)	25.5	42.1	90
HIC-YOLOv5 [43]	(Tang et al., 2023)	26.0	44.3	
YOLO-DCTI [41]	(Min et al., 2023)	27.4	49.8	15
Drone-YOLO-L [6]	(Zhang et al., 2023)	31.9	51.3	
Deformable-DETR [18]	(Zhu et al., 2020)	27.1	43.1	19
RT-DETR-R18 [20]	(Zhao et al., 2023)	27.7	45.8	76
Ours		33.9	53.9	30

**Table 3:** Comparative results before and after model improvement on VisDrone2019-test.

Method	mAP^50–95^/%	mAP^50^/%	mAP^S^/%	mAP^M^/%	mAP^L^/%
RT-DETR	20.7	36.2	11.6	29.4	34.8
Drone-DETR	24.9	42.4	17.8	35.3	40.6

**Table 4:** Comparative results before and after model improvement on DOTA dataset.

Method	mAP^50–95^/%	mAP^50^/%	mAP^S^/%	mAP^M^/%	mAP^L^/%
RT-DETR	41.7	65.1	21.1	44.0	51.6
Drone-DETR	43.2	66.7	24.1	45.2	52.2

**Table 5:** Comparison test of Fast-Residual Block vs Residual Block on VisDrone2019-Val.

Method	mAP^50^/%	Params/M	GFLOPs	Latency/ms
Baseline	45.8	20.1	58.3	13.12
Figure 12b	45.6	17.3	53.3	12.69
Figure 12c	45.7	16.6	48.3	10.56
Figure 12d	45.9	16.5	43.6	10.21

**Table 6:** Performance comparison of different models on VisDrone2019 (test-dev and Val).

Dataset	Experiment	Baseline	ESDNet	EDF-FAM	P2 Layer	mAP^50^/%	Params/M	GFLOPs	FPS
Test	A	√	-	-	-	36.2	20.1	58.3	76
	B	√	√	-	-	37.3	13.8	71.4	47
	C	√	√	√	-	39.7	22.1	67.9	52
	D	√	√	√	√	42.4	28.7	128.3	30
Val	A	√	-	-	-	45.8	20.1	58.3	76
	B	√	√	-	-	47.2	13.8	71.4	47
	C	√	√	√	-	49.4	22.1	67.9	52
	D	√	√	√	√	53.9	28.7	128.3	30

## Data Availability

We will update the training source code and pre-trained models at https://github.com/Ame1999c/Drone-DETR (accessed on 5 June 2024).
